# Menopausal hormone therapy increases the risk of gallstones: Health Insurance Database in South Korea (HISK)-based cohort study

**DOI:** 10.1371/journal.pone.0294356

**Published:** 2023-12-04

**Authors:** Jin-Sung Yuk, Ji Young Park

**Affiliations:** 1 Department of Obstetrics and Gynecology, Sanggye Paik Hospital, School of Medicine, Inje University, Seoul, Republic of Korea; 2 Department of Internal Medicine, Sanggye Paik Hospital, School of Medicine, Inje University, Seoul, Republic of Korea; E-Da Cancer Hospital, TAIWAN

## Abstract

**Objective:**

To determine whether menopausal hormone therapy (MHT) increases the risk of gallstones and gallbladder cancer.

**Design:**

A retrospective cohort study.

**Patients or other participants:**

Data from the Korea National Health Insurance Corporation was obtained between January 1, 2002, and December 31, 2019.

**Interventions:**

Participants were divided into MHT and non-MHT groups; the MHT group was analyzed in detail by dividing participants into tibolone, combined estrogen plus progestin by the manufacturer (CEPM) or physician (CEPP), oral estrogen alone, and topical estrogen subgroups.

**Main outcome measures:**

The incidence of gallstones and gallbladder cancer was compared between the two groups.

**Results:**

This study enrolled 1,004,034 and 381,711 patients in the non-MHT and the MHT groups, respectively. The incidence of gallstones was 2.6% in the non-MHT group and 3.4%, 2.6%, 3.4%, 3.2%, and 4.4% in the tibolone, CEPM, oral estrogen alone, CEPP, and topical estrogen groups, respectively. Cox proportional hazard analysis revealed that all hormones increased the risk of gallstones ([tibolone] hazard ratio [HR]: 1.347, 95% confidence interval [CI]: 1.309–1.387, [CEPM] HR: 1.146, 95% CI: 1.1–1.19, [oral estrogen alone] HR: 1.241, 95% CI: 1.18–1.305, [CEPP] HR: 1.164, 95% CI: 1.01–1.341, [topical estrogen] HR: 1.602, 95% CI: 1.295–1.983). However, the risk of gallbladder cancer did not change with any hormone therapy.

**Conclusions:**

All types of MHT including tibolone, increased the risk of gallstones. This risk was the highest with topical estrogen, which may be a result of selection bias due to concerns regarding the adverse effects of CEE and MPA.

## Introduction

Gallstone disease is a principal epidemiological and economic problem worldwide, with a prevalence of approximately 10%-15% in adults in western countries, 3%-10% in Asian countries and 2%-5% in Korea [1–3]. In Western countries, approximately 75%-80% of gallstones are cholesterol-based and are known to be related to several factors such as metabolic diseases, dietary factors, and hormone replacement therapy [2–5]. Gallstones formation is believed to require changes in three physiological processes—enhanced saturation of biliary cholesterol, more rapid precipitation of cholesterol monohydrated crystals in bile, and decreased contractility of the gallbladder [1, 6].

Estrogen binds to the hepatic estrogen receptor and increases the amount of cholesterol synthesized in the liver, thereby increasing the amount of cholesterol in bile [7–9], while progesterone causes cholestasis by impairing contraction of the smooth muscle of the gallbladder [9]. Several studies have demonstrated that postmenopausal hormone therapy increases the risk of gallbladder disease [6, 8, 10–14]. The follow-up reports of the Heart and Estrogen/progestin Replacement Study (HERS) revealed that females who received hormone therapy were at a 70% increased risk of undergoing cholecystectomy [13, 14], while the Women’s Health Initiative (WHI) study reported that the incidence of gallbladder disease increased in patients who used conjugated equine estrogen (CEE) plus medroxyprogesterone acetate (MPA) as well as in patients who used CEE alone [10]. However, the CEE plus MPA regimen used in previous studies was found to induce breast cancer, therefore, it is rarely prescribed in clinical practice [10, 11]. Additionally, although the risks and benefits of hormone therapy differed depending on the age or menopause period of the patient in previous studies, the participants of these studies were relatively old [1, 10, 14]. Moreover, the study population in such a previous study comprised several highly obese participants with a body mass index (BMI) of ≥ 30 kg/m^2^ [11]. Therefore, the effects of age and obesity, known as the single risk factors for gallstone development, were not considered in previous studies.

Therefore, this study aimed to evaluate the risk of gallstone disease and gallbladder cancer following the use of menopausal hormone therapy (MHT) using Korea’s national health claim data. In particular, this study analyzes tibolone, which has not been studied much in previous studies but has a high prescription rate in Korea.

## Materials and methods

### Database

This was a retrospective cohort study based on national population data from the National Health Insurance Service (NHIS) of Korea obtained between January 1, 2002 and December 31, 2019. Korea’s health insurance is integrated into a single system operated by the National Health Insurance Corporation, and most people living in Korea are enrolled under National Health Insurance. Therefore, health insurance information (such as details on age, gender, diagnosis, surgery name, and prescriptions) is included in the database. Additionally, National Cancer Screening Program was introduced as part of the national cancer management plan to provide free screening for stomach, colorectal, liver, breast, and cervical cancer to individuals based on age. Through screening, the National Health Insurance Corporation includes additional, self-questionnaire data on past and social history, along with cancer screening results. The diagnosis was recorded in the database using the International Classification of Diseases, 10^th^ revision (ICD-10), and surgery and procedures were recorded using Korea Health Insurance Medical Care Expenses (2012, 2016, and 2019 versions).

### Participants selection

To identify the case and control groups in this study, only females aged >40 years who were undergoing menopause between 2002 and 2011 (at the time of interview) were selected. Women with recorded menopause in 2002, menopause before the age of 40 years, or a diagnostic code for any type of cancer (diagnostic code: Cxx) or gallbladder disease (diagnostic code: K80-83, K87), >180 days from the start date were excluded. The start date for the MHT group was defined as the day on which the examination was performed, if only the examination year was recorded, June 30 of the examination year was defined as the start date. In the absence of other events, the last study date was set as the date of death or December 31, 2019. The MHT group only included females who used at least one MHT for at least 6 months between 2002 and 2011. The non-MHT group included females who had never been prescribed MHT drugs between 2002 and 2019.

### Outcomes

Gallstones were diagnosed when the patient visited a medical institution with the gallstone diagnostic code K80 at least three times. Cholecystitis was diagnosed when the patient visited a medical institution with the cholecystitis diagnostic code K81 at least three times. Gallbladder cancer was defined as three or more visits to a medical institution with the gallbladder or biliary tract cancer diagnostic codes C23 and C24.

### Variables

In this study, menopausal hormones were limited to tibolone, combined estrogen plus progestin by the manufacturer (CEPM), oral estrogen alone, combined estrogen plus progestin by the physician (CEPP), and topical estrogen. A detailed list of medications is presented in S1 Table. If two or more menopausal hormone treatments were used sequentially, patients were assigned to the group associated with the last menopausal hormone treatment used for more than 6 months. The reference date for age, BMI, region, socioeconomic status (SES), Charlson Comorbidity Index (CCI), age of menarche, age at menopause, parity, smoking status, drinking history, amount of physical exercise, and the period from menopause to participant inclusion was defined as the study participation date. In this study, obesity (according to BMI) was defined as per the standards of the Asia-Pacific perspective (≥ 25 kg/m^2^). A low SES was defined when the type of medical insurance was medical aid. The urban area was defined when the location of the medical institution that provided prescriptions or performed the examinations was metropolitan. The CCI was calculated using the diagnostic codes in the medical insurance records from 1 year before the study participation date to the actual study participation date. Smoking history was classified as “never”, “past”, and “current”, and drinking history was classified according to the number of drinks consumed per week. Exercise strength was determined according to the frequency of exercise for 30 minutes or more per week.

### Statistical analysis

All statistical data were subjected to a two-sided test, and a p-value of <0.05 was considered statistically significant. All continuous variables are presented as median [25^th^, 75^th^ percentile], and all categorical variables are presented as number (percentage). In this study, the Cox proportional hazard model was used to determine the risk of gallbladder disease. As a sensitivity test to confirm robustness, only cases wherein MHT was prescribed by an obstetrician and gynecologist were selected and analyzed. Listwise deletion was implemented for the treatment of missing values and all statistical analyses were performed using SAS Enterprise Guide 6.1 (SAS Institute, Cary, North Carolina, United States).

### Ethics

This study was approved by the Institutional review board of Inje University Sanggye Paik Hospital (approval number SAPAIK-2020-08-0002). Information that could identify individuals was removed according to the privacy policy of the NHIS, and analysis was only possible using a virtual server of the NHIS. Therefore, the researcher was only able to export results for the thesis ensuring that no harm was caused to the individuals participating in the study. According to the Bioethics and Safety Act of South Korea, it was not necessary to obtain informed consent from the participants of this study.

## Results

**Fig 1:**
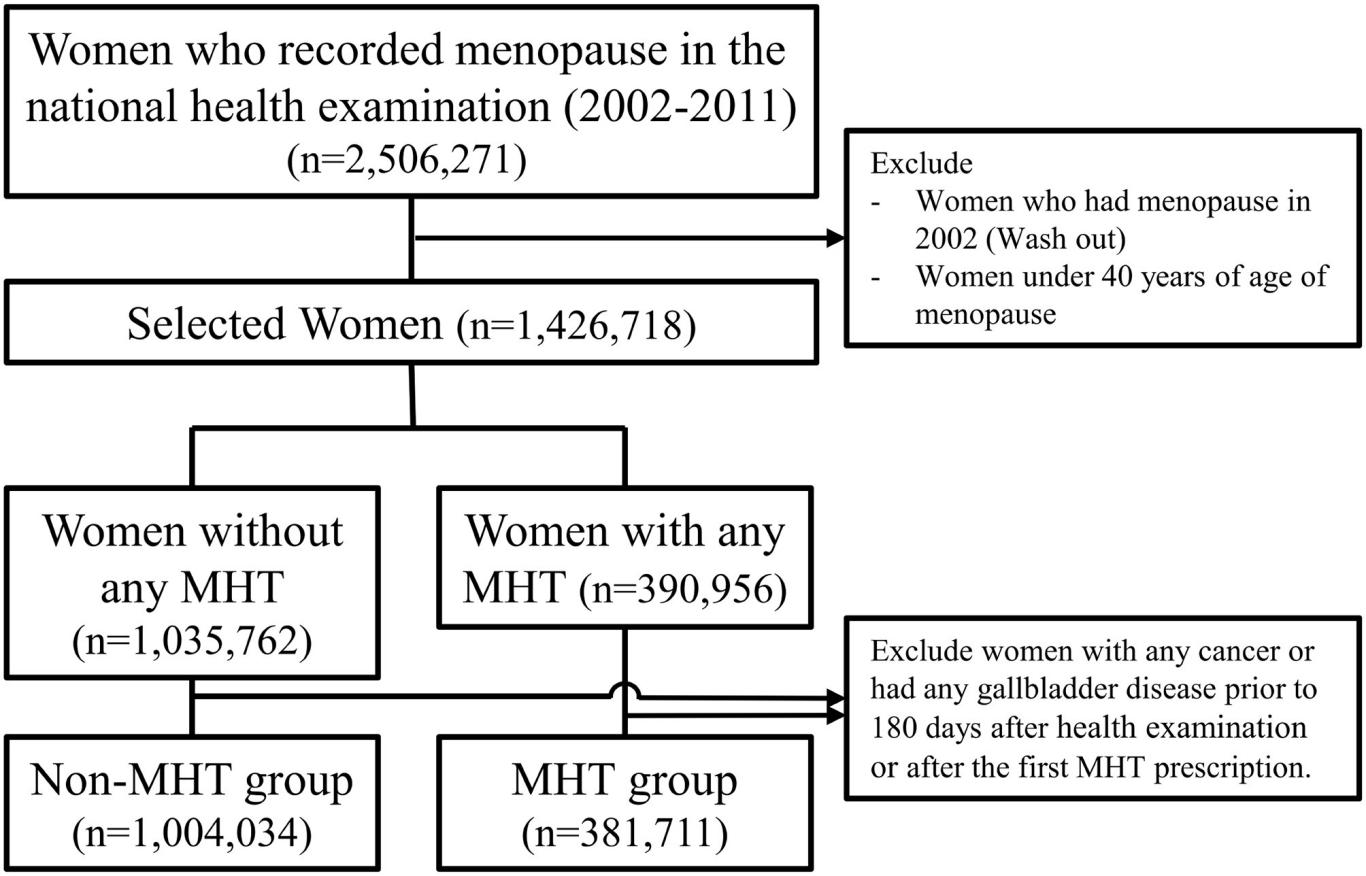
Flowchart to select to participants according to the use of menopausal hormones in this study. MHT, Menopausal hormone therapy.

A total of 2,506,271 individuals were collected from January 1, 2002 to December 31, 2019, of which 1,004,034 from the non-MHT group and 381,711 from the MHT group were ultimately selected for this study and the average age was 56 [52–62] years (Fig 1). The follow-up period was 11.5 [9.5–13.5] years and 12.7 [10.4–14.9] years for the MHT and non-MHT groups, respectively. In the MHT group, tibolone, CEPM, oral estrogen alone, CEPP, and topical estrogen were used in 192,671, 128,168, 52,404, 6,427, and 2,041 patients, respectively. The characteristics of the study participants are detailed in Table 1. The incidence of gallstones was 26,548 (2.6%) in the non-MHT group, 6,540 (3.4%) in the tibolone group, 3,369 (2.6%) in the CEPM group, 1,756 (3.4%) in the oral estrogen alone group, 205 (3.2%) in the CEPP group, and 90 (4.4%) in the topical estrogen group. The incidence of gallbladder cancer was 1,686 (0.2%) in the non-MHT group, 238 (0.1%) in the tibolone group, 124 (0.1%) in the CEPM group, 77 (0.1%) in the oral estrogen alone group, 77 (0.1%) in the CEPP group. 6 (0.1%), and 6 (0.3%) in the topical estrogen group. In the MHT group, the duration of hormone therapy use was 25 [11–58] months, 25 [11–58] months, 15 [9–40] months, 16 [9–35] months, and 13 [8–24] months, respectively, for the tibolone, CEPM, oral estrogen alone, CEPP, topical estrogen subgroups. Table 2 shows the detailed characteristics of hormone prescription in the MHT group.

**Table 1:** Characteristics of participating women according to each MHT use.

	Non-MHT	Tibolone	Combined Estrogen plus progestin by the manufacturer	Oral Estrogen	Combined Estrogen plus progestin by the physician	Topical estrogen	Total
Nunber of women	1,004,034	192,671	128,168	52,404	6,427	2,041	1,385,745
Median age (years)	58 [52–64]	53 [50–57]	52 [50–56]	53 [49–57]	54 [51–59]	53 [50–57]	56 [52–62]
Age at inclusion (years)							
40~49	84,417 (8.4)	33,788 (17.5)	29,306 (22.9)	13,632 (26)	1,123 (17.5)	430 (21.1)	162,696 (11.7)
50~59	484,104 (48.2)	125,909 (65.3)	85,192 (66.5)	29,244 (55.8)	3,795 (59)	1,250 (61.2)	729,494 (52.6)
60~69	301,557 (34.7)	29,170 (15.4)	12,598 (9.9)	7,794 (15.4)	1,313 (21.1)	326 (16.3)	352,758 (28.3)
70~	133,956 (13.3)	3,804 (2)	1,072 (0.8)	1,734 (3.3)	196 (3)	35 (1.7)	140,797 (10.2)
Median BMI (kg/m^2^)	24 [22.1–26.1]	23.5 [21.8–25.4]	23.1 [21.5–25]	23.7 [22–25.7]	23.3 [21.6–25.3]	23.7 [21.9–25.6]	23.8 [21.9–25.9]
BMI (kg/m2)							
<18.5	18,791 (1.9)	3,309 (1.7)	2,568 (2)	764 (1.5)	133 (2.1)	45 (2.2)	25,610 (1.9)
18.5–22.9	337,118 (34.3)	77,308 (40.5)	57,623 (45.3)	19,199 (37)	2,663 (41.8)	760 (37.5)	494,671 (36.3)
23–24.9	260,332 (26.5)	52,950 (27.8)	34,153 (26.8)	14,541 (28)	1,765 (27.7)	534 (26.4)	364,275 (26.7)
25–29.9	325,102 (33)	52,386 (27.5)	30,410 (23.9)	15,690 (30.2)	1,669 (26.2)	629 (31)	425,886 (31.3)
≥30	42,479 (4.3)	4,828 (2.5)	2,512 (2)	1,762 (3.4)	146 (2.3)	58 (2.9)	51,785 (3.8)
SES							
Mid~high SES	960,866 (95.7)	185,728 (96.4)	124,928 (97.5)	50,919 (97.2)	6,266 (97.5)	1,982 (97.1)	1,330,689 (96)
Low SES	43,168 (4.3)	6,943 (3.6)	3,240 (2.5)	1,485 (2.8)	161 (2.5)	59 (2.9)	55,056 (4)
Region							
Urban area	294,903 (29.4)	59,839 (31.1)	43,287 (33.8)	16,458 (31.4)	3,244 (50.5)	921 (45.1)	418,652 (30.2)
Rural area	709,131 (70.6)	132,832 (68.9)	84,881 (66.2)	35,946 (68.6)	3,183 (49.5)	1,120 (54.9)	967,093 (69.8)
CCI							
0	656,540 (65.4)	130,604 (67.8)	90,490 (70.6)	36,399 (69.5)	4,416 (68.7)	1,327 (65)	919,776 (66.4)
1	199,130 (19.8)	37,813 (19.6)	23,407 (18.3)	9,649 (18.4)	1,219 (19)	383 (18.8)	271,601 (19.6)
≥2	148,364 (14.8)	24,254 (12.6)	14,271 (11.1)	6,356 (12.1)	792 (12.3)	331 (16.2)	194,368 (14)
Parity (years)							
0 or not respond	164,289 (16.4)	29,963 (15.6)	16,060 (12.5)	10,997 (21)	1,267 (19.7)	449 (22)	223,025 (16.1)
1	59,267 (5.9)	16,739 (8.7)	13,332 (10.4)	4,005 (7.6)	471 (7.3)	160 (7.8)	93,974 (6.8)
2	659,839 (74.7)	128,551 (73.4)	89,322 (75.2)	32,210 (68.2)	4,030 (69.9)	1,229 (66.9)	915,181 (74.3)
≥3	120,639 (12)	17,418 (9)	9,454 (7.4)	5,192 (9.9)	659 (10.3)	203 (9.9)	153,565 (11.1)
Age at menarche (years)							
<13	156,222 (15.6)	27,914 (14.6)	18,258 (14.3)	9,606 (18.6)	1,193 (18.7)	379 (18.8)	213,572 (15.5)
≥13	843,102 (84.4)	163,242 (85.4)	109,174 (85.7)	42,125 (81.4)	5,179 (81.3)	1,637 (81.2)	1,164,459 (84.5)
Age at menopause (years)							
40–44	120,211 (12)	23,115 (12)	14,382 (11.2)	11,294 (21.6)	821 (12.8)	402 (19.7)	170,225 (12.3)
45–49	289,518 (28.8)	62,716 (32.6)	42,933 (33.5)	18,587 (35.5)	2,049 (31.9)	738 (36.2)	416,541 (30.1)
50–54	506,064 (55.3)	92,258 (51.8)	62,335 (52.1)	19,965 (40.1)	3,039 (51.4)	777 (40.5)	684,438 (53.8)
55-	88,241 (8.8)	14,582 (7.6)	8,518 (6.6)	2,558 (4.9)	518 (8.1)	124 (6.1)	114,541 (8.3)
Smoking							
Never	915,250 (96.4)	173,854 (93.8)	116,024 (93.5)	47,863 (95)	5,923 (95.6)	1,876 (96.3)	1,260,790 (95.7)
Past	9,652 (1)	3,162 (1.7)	2,264 (1.8)	703 (1.4)	85 (1.4)	30 (1.5)	15,896 (1.2)
Current	24,678 (2.6)	8,380 (4.5)	5,801 (4.7)	1,813 (3.6)	185 (3)	43 (2.2)	40,900 (3.1)
Alcohol (per week)							
None	814,065 (85.4)	144,807 (77.6)	94,626 (75.8)	40,485 (79.7)	5,160 (82.7)	1,627 (82.3)	1,100,770 (83.2)
~2/week	119,064 (12.5)	35,528 (19)	25,781 (20.7)	8,948 (17.6)	951 (15.2)	310 (15.7)	190,582 (14.4)
3~6/week	14,736 (1.6)	4,853 (2.6)	3,523 (2.8)	971 (1.9)	90 (1.5)	30 (1.5)	24,203 (1.8)
Daily	5,325 (0.6)	1,492 (0.8)	900 (0.7)	375 (0.7)	39 (0.6)	10 (0.5)	8,141 (0.6)
Physical exercise (per week)							
None	626,662 (65.6)	110,995 (59.5)	74,801 (59.9)	30,285 (59.7)	3,563 (57.1)	1,036 (52.7)	847,342 (63.9)
1~2	156,356 (16.4)	35,585 (19.1)	24,376 (19.5)	9,746 (19.2)	1,220 (19.6)	426 (21.7)	227,709 (17.2)
3~4	86,645 (9.1)	21,377 (11.5)	14,543 (11.6)	5,609 (11.1)	804 (12.9)	289 (14.7)	129,267 (9.8)
5~6	28,987 (3)	7,032 (3.8)	4,764 (3.8)	1,785 (3.5)	239 (3.8)	84 (4.3)	42,891 (3.2)
Daily	56,123 (5.9)	11,570 (6.2)	6,361 (5.1)	3,316 (6.5)	410 (6.6)	131 (6.7)	77,911 (5.9)
Period from menopause to inclusion (years)							
<5	388,428 (38.7)	111,808 (58)	87,230 (68.1)	27,330 (52.2)	3,306 (51.4)	1,039 (50.9)	619,141 (44.7)
5~9	209,061 (20.8)	44,237 (23)	25,390 (19.8)	13,075 (25)	1,541 (24)	538 (26.4)	293,842 (21.2)
10~	406,545 (40.5)	36,626 (19)	15,548 (12.1)	11,999 (22.9)	1,580 (24.6)	464 (22.7)	472,762 (34.1)

BMI, Body mass index, CCI, Charlson comorbidity index; MHT, menopausal hormone therapy; SES, socioeconomic status

Data are expressed as the number (%) or median [25th percentile, 75th percentile].

**Table 2:** Characteristics about women who have menopausal hormone.

MHT chracteristics	Tibolone	Combined Estrogen plus progestin by manufacturer	Oral Estrogen	Combined Estrogen plus progestin by physician	Topical estrogen	Total MHT
Median duration (months)	25 [11–58]	25 [11–58]	15 [9–40]	16 [9–35]	13 [8–24]	23 [10–55]
Duration (years)						
<5	145,461 (75.5)	97,060 (75.7)	43,376 (82.8)	5,597 (87.1)	1,943 (95.2)	293,437 (76.9)
5–9.9	34,634 (18)	23,786 (18.6)	6,280 (12)	641 (10)	93 (4.6)	65,434 (17.1)
≥10	12,576 (6.5)	7,322 (5.7)	2,748 (5.2)	189 (2.9)	5 (0.2)	22,840 (6)
Duration of previous other MHT (years)						
<5	187,369 (97.2)	126,104 (98.4)	51,635 (98.5)	5,352 (83.3)	2,016 (98.8)	372,476 (97.6)
5–9.9	4,737 (2.5)	1,895 (1.5)	691 (1.3)	799 (12.4)	24 (1.2)	8,146 (2.1)
≥10	565 (0.3)	169 (0.1)	78 (0.1)	276 (4.3)	1 (0)	1,089 (0.3)
Last dosage of Tibolone (per day)						
1.25mg	1,819 (0.9)					
2.5mg	190,658 (99)					
over 5mg	175 (0.1)					
Prescribed specialty						
Gynecology	64,852 (33.7)	58,694 (45.8)	21,624 (41.3)	1,532 (23.8)	510 (25)	147,212 (38.6)
Non-gynecology	127,819 (66.3)	69,474 (54.2)	30,780 (58.7)	4,895 (76.2)	1,531 (75)	234,499 (61.4)

MHT, menopausal hormone therapy

Data are expressed as the number (%) or median [25th percentile, 75th percentile].

**Fig 2:**
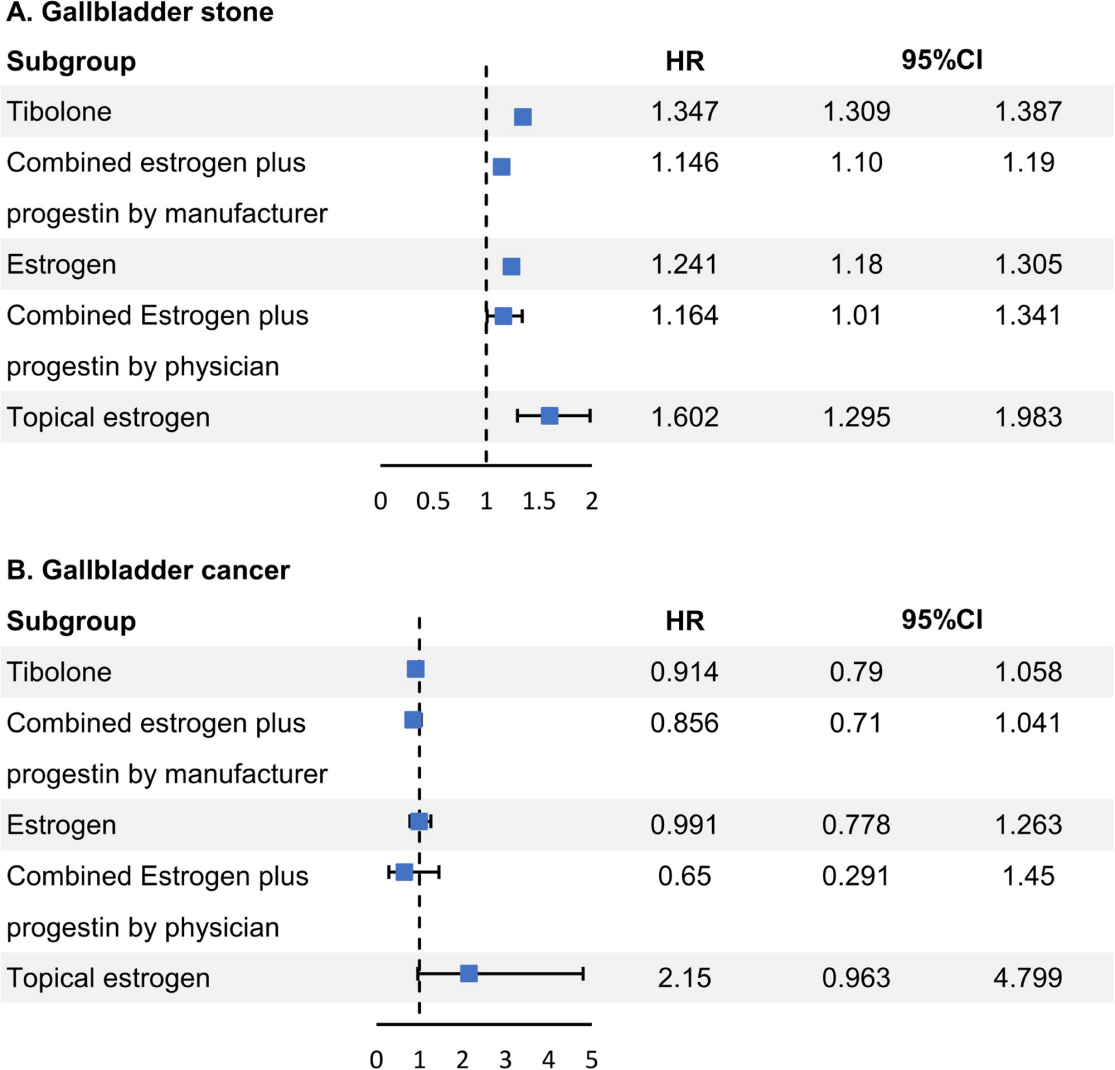
Risk of gallstone and gallbladder cancer according to the types of menopausal hormones. HR, Hazard ratio; CI, confidence interval.

Cox proportional hazard analysis with all variables adjusted revealed that all hormones increased the incidence of gallstones ([tibolone] hazard ratio [HR]: 1.347, 95% confidence interval [CI]: 1.309–1.387, [CEPM] HR: 1.146, 95% CI: 1.1–1.19, [oral estrogen alone] HR: 1.241, 95% CI: 1.18–1.305, [CEPP] HR: 1.164, 95% CI: 1.01–1.341, [topical estrogen] HR: 1.602, 95% CI: 1.295–1.983) (Fig 2). However, no significant change was noted in the risk of gallbladder cancer for all hormonal drugs ([tibolone] HR: 0.914, 95% CI: 0.79–1.058, [CEPM] HR: 0.856, 95% CI: 0.71–1.041, [oral estrogen alone] HR: 0.991, 95% CI: 0.778–1.263, [CEPP] HR: 0.65, 95% CI: 0.291–1.45, [topical estrogen] HR: 2.15, 95% CI: 0.963–4.799). An increased risk of gallstones was observed in patients aged >70 years (HR: 1.498, 95% CI: 1.394–1.603), patients with a BMI ≥30 kg/m^2^ (severely obese; HR: 1.824, 95% CI: 1.739–1.913), patients with a low SES (HR: 1.38, 95% CI: 1.304–1.46), current smoker (HR: 1.261, 95% CI: 1.192–1.334), and those ≥10 years after menopause (HR: 1.166, 95% CI: 1.114–1.219). Conversely, the risk of gallstones decreased in rural areas (HR: 0.94, 95% CI: 0.919–0.962) and in patients who consumed drink 3–6 times per week (HR: 0.899, 95% CI: 0.825–0.979) (S1 Table).

The risk of gallbladder cancer increased with age and obesity, regardless of MHT (S2 Table). In a subgroup analysis of patients using only tibolone, no significant difference was noted in the dose of tibolone (Table 3). In a subgroup analysis according to age, the effects of gallbladder disease according to the effects of hormones were mostly affected in patients in their 50s and generally did not affect patients aged ≥60 years (S3 and S4 Tables).

**Table 3:** Analysis of subgroups in terms of the risk of gallbladder disease in accordance with the primary variables in tibolone.

Tibolone use	HR (95% CI) ^a^	P-value
Tibolone only (without non-MHT)		
Period from menopause to inclusion (years)		
5~9	1.063 (1.003–1.128)	0.041
10~	1.149 (1.05–1.259)	0.003
Total period of use (months)	1.002 (1.001–1.002)	<0.001
Dosage		
1.25mg	1.054 (0.858–1.295)	0.615
Over 5mg	1.043 (0.468–2.322)	0.918
Prescribed specialty		
Non-gynecology	1.042 (0.997–1.09)	0.07
Dosage of tibolone		
Tibolone 1.25mg vs Non-MHT	1.315 (1.072–1.614)	0.009
Tibolone 2.5mg vs Non-MHT	1.245 (1.215–1.275)	<0.001
Tibolone 5mg vs Non-MHT	1.117 (0.735–1.697)	0.606

CI, confidence interval; HR, hazard ratio; MHT, menopausal hormone therapy

^a^ HRs were adjusted for age group, body mass index, socioeconic status, region, Charlson comorbidity index, parity, age at menarche, age at menopause, smoking, alcohol, physical exercise, period from menopause to inclusion.

## Discussion

In this study, we examined if MHT increases the risk of gallstones and gallbladder cancer and found that the risk of gallstones increased for all hormones in the MHT group. In addition to CEE plus MPA, which has been previously studied, tibolone reportedly increased the risk of gallstones [15]. Tibolone is a compound that exerts estrogenic, progestogenic, and androgenic properties and is known to have a beneficial effect on menopause symptoms [15, 16]. An adverse androgenic effect of tibolone is that it alters the lipid profile [15, 16], and although the exact mechanism is unclear, previous studies have reported that tibolone alters the cholesterol metabolism in the liver [15]. Czerny et al. demonstrated that tibolone increases cholesterol level in bile, however, few other studies have established a relationship between tibolone and gallstone formation [15]. In particular, estrogen has been shown to increase the risk of gallstones in many previous studies [6–8, 17, 18]. In the WHI study, CEE increased the risk of gallstones regardless of age [10], however, after the study, the use of combined estrogen and progestin therapy decreased as a postmenopausal hormone therapy as it was found to increase the risk of breast cancer [10, 11]. In the same study, although estrogen monotherapy relatively lowered the risk of breast cancer, the risk was unclear in the late post-intervention follow-up period, thus, oral estrogen use was also reduced [10, 11].

In this study, topical estrogen was associated with the highest risk of gallstones when compared with other hormonal agents. This is in contrast with results from previous studies regarding the risk of gallstones according to the estrogen administration method [19]. This result may be due to the selection bias of prescribing more topical estrogen to patients at a high risk of gallstones due to other previous studies on oral estrogen [19]. Another previous study investigated whether transdermal estrogen could lower the risk of gallstones compared with oral estrogen and revealed that transdermal estrogen lowered the cholesterol saturation in the bile as well as the risk of gallstones [20]. This is because oral estrogen enhances the liver action on lipid and protein synthesis compared to transdermal estrogen [20–23]. Orally administered estrogen enters the systemic circulation after being metabolized in the liver, with its metabolites excreted in bile or urine [22], however, transdermally administered estrogen bypasses the metabolic process in the liver and enters the systemic circulation through the skin [20–24]. According to a study published by Meike et al., oral CEE significantly altered liver markers such as lipids and sex hormone-binding globulin than transdermal estrogen [19]. However, both oral and transdermal administration of estrogen resulted in a significant increase in the biliary saturation of cholesterol and decrease in the time required to form cholesterol crystals in the bile thus, estrogen increased the likelihood of gallstone formation regardless of the mode of administration [19].

None of the hormonal treatments administered in this study increased the risk of gallbladder cancer. Gallbladder cancer is only cancer that occurs more frequently in women among biliary tract cancers [9, 12, 25], and this pronounced female predominance suggests that sex hormones are involved in the development of gallbladder cancer [9, 12, 25, 26]. Several observational studies have demonstrated that the incidence of gallbladder cancer correlates with old age, obesity, rapid weight change, and female sex; among females, gallbladder cancer is related to female reproductive factors such as parity and age at the birth of the first child [9, 12, 27–29]. Although the relationship between sex hormones and gallbladder cancer has not been clearly elucidated, there is a hypothesis that estrogen acts independently on gallbladder carcinogenesis, additionally, it is hypothesized that estrogen may indirectly influence the development of gallbladder cancer by increasing the incidence of gallstones [25, 30, 31]. Several studies regarding the relationship between the use of MHT and the development of gallbladder cancer have shown that MHT use increases the risk of gallbladder cancer [12, 25, 29], however, other studies have demonstrated opposite results [26]. In a study published by Kilander et al., the incidence of gallbladder cancer was reduced in the MHT group [26]. This may be due to the increased incidence of gallstones in the MHT group (which included many patients who underwent cholecystectomy) or the small number of patients with gallbladder cancer among the study participants, and short follow-up period [26]. Similarly, the number of patients with gallbladder cancer in our study was small (total: 2,137 [0.2%]; non-MHT: 1,686 [0.2%]; MHT: 451 [0.1%]), and additional analyses such as analysis according to hormone treatment dose were not performed. Moreover, the study did not correct for patients who had already undergone cholecystectomy. Therefore, the relationship between MHT and gallbladder cancer needs to be further analyzed in detail. In our study, age and obesity, which are known risk factors for gallbladder cancer [30, 32], were also found to be relevant to gallbladder cancer. Therefore, regardless of MHT, the risk of gallbladder cancer increased with older age and higher BMI. The study by Chen et al. have focused on the association between gallbladder disease and metabolic syndrome, as obesity, diabetes, and hyperlipidemia, which are known risk factors for gallbladder disease, match the criteria for metabolic syndrome [33]. Further evaluation of the impact of metabolic syndrome on the relationship between MHT and the development gallbladder disease is needed.

The strength of this study is that it is a large-scale Asian population study with more than 1 million participants, and the number of study participants is sufficiently comparable to that of previously published observational studies. Additionally, most variables that could not be partially corrected for in existing observational studies were corrected in this study, including age, BMI, SES, CCI, age of menarche, age at menopause, parity, smoking status, drinking history, amount of physical exercise, and the period from menopause to inclusion. The prescription rate of tibolone was the highest among included hormones, and tibolone is currently the most prescribed MHT in Korea. Tibolone is widely used to relieve menopausal symptoms due to concerns regarding the adverse effects of CEE or CEE plus MPA. However, as tibolone was not a mainstream prescription drug in previous studies [8, 22], few studies have been conducted regarding this hormone. In this study, tibolone and various CEPMs (Angeliq^®^, Climen^®^, Clian^®^, or Femoston^®^) were included. However, the limitation of this study is that the detailed drug list constituting the CEPM group could not be confirmed due to the NHIS policy, and additional research is required. Another limitation of this study is that MHT and non-MHT groups were different in sizes, which could introduce statistical biases. This may require further correction.

Our large cohort study confirmed that MHT increased the risk of developing gallstones. It was particularly meaningful to find out that tibolone, which has been rarely studied before, increases the risk of gallstones. Contrary to findings of previous studies, we found that topical estrogen increased the incidence of gallstones when compared with other estrogen agents, however, additional research is needed to confirm this observation. Additionally, when prescribing hormone therapy, these results should be carefully considered. While MHT and gallbladder cancer was not found to correlate in this study, few studies regarding exogenous estrogen administration and gallbladder cancer have been published to date, and existing studies have different results. Therefore, additional large-scale studies are needed in this regard.

## Supporting information

S1 Table Menopausal hormones are organized into groups. (PDF)

S2 Table Hazard ratios for determining the likelihood of developing gallbladder disease based on the major variables. (PDF)

S3 Table The gallbladder disease cases per 100,000 person-years in this research. (PDF)

S4 Table Statistical analysis about the risk of gallbladder disease in relation to age. (PDF)
