# Supplementary material for: Menopausal hormone therapy increases the risk of gallstones: Health Insurance Database in South Korea (HISK)-based cohort study
Source: PLoS One. 2023 Dec 4;18(12):e0294356. doi: 10.1371/journal.pone.0294356 (PMC10695378; doi:10.1371/journal.pone.0294356)
Supplement: S1 Table — (PDF) [file pone.0294356.s001.pdf]

Supplementary table 1. Menopausal hormones are organized into groups.

MHT (generic name)	Brand name
Combined Estrogen plus progestin by the manufacturer	
Estradiol Hemihydrate 1.03mg, Drospirenone 2mg	Angelic
Estradiol Valerate 2mg, Medroxyprogesterone Acetate 10mg	Divina
Estradiol Valerate 1mg, Medroxyprogesterone Acetate 2.5mg	Indivina Tab 1mg/2.5mg
Estradiol Valerate 1mg, Medroxyprogesterone Acetate 5mg	Indivina Tab 1mg/5mg
Estradiol Valerate 2mg, Medroxyprogesterone Acetate 2.5mg	Indivina Tab 2mg/2.5mg
Estradiol Hemihydrate 2mg, Norethisterone Acetate 1mg	Cliane
Estradiol Hemihydrate 2.06mg, Dydrogesterone 10mg	Femoston 2/10
Estradiol Hemihydrate 1.03mg, Dydrogesterone 10mg	Femoston 1/10
Estradiol Hemihydrate 1.03mg, Dydrogesterone 5mg	Femoston Coti
Cyproterone Acetate 1mg, Estradiol Valerate 2mg	Climen
Estradiol Hemihydrate 1.03mg, Norethisterone Acetate 0.5mg	Esdiol-half
Estradiol Valerate 1.31mg, Norethisterone Acetate 0.5mg	Cliovelle
Combined Estrogen plus progestin by the physician [a]	
Progesterone Micronized 100mg	Utrogestan 100mg
Medroxyprogesterone Acetate 5mg	Provera 5mg
Medroxyprogesterone Acetate 10mg	Provera 10mg
Dydrogesterone 10mg	Duphaston
Estrogen	
Conjugated Estrogens 0.3mg	Premina 0.3mg
Conjugated Estrogens 0.625mg	Premina 0.625mg
Estradiol Valerate 1mg	Progynova 1mg
Estradiol Valerate 2mg	Progynova 2mg
Estradiol Hemihydrate 1mg	Preda 1mg

Tibolone	
Tibolone 2.5mg	Livial, Rabilone, Libolone, Libron, Live on, Liviem, Tiborisi, Tibolan, Tibiol, Pharmbio Korea Tibolone, Hyundai Tibolone
Topical estrogen	
Estradiol Hemihydrate	Estreva Gel, Climara patch, Divigel Gel

MHT, menopausal hormone therapy

[a] This group used the progestin below and the estrogen group above simultaneously.
