# Supplementary material for: Menopausal hormone therapy increases the risk of gallstones: Health Insurance Database in South Korea (HISK)-based cohort study
Source: PLoS One. 2023 Dec 4;18(12):e0294356. doi: 10.1371/journal.pone.0294356 (PMC10695378; doi:10.1371/journal.pone.0294356)
Supplement: S2 Table — (PDF) [file pone.0294356.s002.pdf]

Supplementary table 2. Hazard ratios for determining the likelihood of developing gallbladder disease based on the major variables.

Variables	Gallballder stone [a]		Cholecystitis [a]		Gallballder cancer [a]	
	HR (95% CI) [a]	P-value	HR (95% CI) [a]	P-value	HR (95% CI) [a]	P-value
MHT						
Tibolone	1.347 (1.309-1.387)	<0.001	1.232 (1.155-1.315)	<0.001	0.914 (0.79-1.058)	0.23
Combined estrogen plus progestin by the manufacturer	1.146 (1.103-1.19)	<0.001	1.15 (1.058-1.251)	0.001	0.856 (0.705-1.041)	0.119
Oral Estrogen	1.241 (1.18-1.305)	<0.001	0.981 (0.87-1.107)	0.756	0.991 (0.778-1.263)	0.941
Combined Estrogen plus progestin by the physician	1.164 (1.01-1.341)	0.036	1.024 (0.74-1.415)	0.887	0.65 (0.291-1.45)	0.293
Topical estrogen	1.602 (1.295-1.983)	<0.001	0.962 (0.532-1.739)	0.898	2.15 (0.963-4.799)	0.062
Age at inclusion (years)						
50~59	1.074 (1.031-1.119)	<0.001	1.082 (0.988-1.186)	0.091	1.715 (1.357-2.166)	<0.001
60~69	1.198 (1.127-1.272)	<0.001	1.377 (1.207-1.572)	<0.001	2.807 (2.089-3.774)	<0.001
70~	1.495 (1.394-1.603)	<0.001	2.234 (1.926-2.59)	<0.001	4.801 (3.482-6.621)	<0.001
BMI (kg/m2)						
<18.5	0.906 (0.828-0.992)	0.033	0.954 (0.796-1.144)	0.613	0.756 (0.502-1.137)	0.179
23-24.9	1.187 (1.155-1.221)	<0.001	1.06 (0.998-1.126)	0.057	1.072 (0.95-1.209)	0.26
25-29.9	1.393 (1.357-1.429)	<0.001	1.242 (1.175-1.313)	<0.001	1.268 (1.136-1.415)	<0.001
≥30	1.824 (1.739-1.913)	<0.001	1.557 (1.404-1.727)	<0.001	1.342 (1.081-1.666)	0.008
SES						
Low SES	1.38 (1.304-1.46)	<0.001	1.444 (1.285-1.624)	<0.001	0.973 (0.734-1.292)	0.852
Region						
Rural area	0.94 (0.919-0.962)	<0.001	1.032 (0.982-1.085)	0.214	1.026 (0.929-1.134)	0.608
CCI						
1	1.109 (1.08-1.139)	<0.001	1.207 (1.142-1.276)	<0.001	1.049 (0.938-1.173)	0.4

≥2	1.272 (1.236-1.309)	<0.001	1.328 (1.249-1.412)	<0.001	1.091 (0.961-1.237)	0.178
Parity (years)						
0	0.975 (0.924-1.027)	0.339	1.023 (0.908-1.153)	0.712	1.083 (0.839-1.399)	0.54
2	0.948 (0.907-0.991)	0.018	1.07 (0.966-1.184)	0.195	1.127 (0.9-1.41)	0.297
≥3	1.019 (0.968-1.073)	0.472	1.016 (0.904-1.142)	0.79	1.095 (0.854-1.405)	0.472
Age at menarche (years)						
≥13	1.025 (0.991-1.062)	0.155	1.009 (0.935-1.088)	0.823	1.067 (0.915-1.243)	0.41
Age at menopause (years)						
45-49	0.985 (0.949-1.023)	0.437	1.033 (0.954-1.119)	0.426	1.177 (0.998-1.388)	0.053
50-54	0.991 (0.951-1.032)	0.656	1.035 (0.949-1.128)	0.434	1.106 (0.93-1.315)	0.254
55-	1.065 (1.006-1.127)	0.031	1.065 (0.944-1.202)	0.307	1.299 (1.029-1.641)	0.028
Smoking						
Past	1.145 (1.044-1.255)	0.004	1.275 (1.054-1.544)	0.013	1.082 (0.709-1.651)	0.715
Current	1.261 (1.192-1.334)	<0.001	1.442 (1.285-1.619)	<0.001	1.025 (0.775-1.356)	0.862
Alcohol (per week)						
~2/week	0.927 (0.898-0.957)	<0.001	0.994 (0.928-1.064)	0.862	1.023 (0.888-1.179)	0.752
3~6/week	0.899 (0.825-0.979)	0.014	1.019 (0.851-1.219)	0.842	1.31 (0.92-1.865)	0.135
Daily	1.007 (0.885-1.146)	0.916	0.958 (0.718-1.277)	0.769	1.366 (0.821-2.274)	0.23
Physical exercise (per week)						
1~2	0.993 (0.965-1.022)	0.63	0.912 (0.856-0.973)	0.005	1.05 (0.927-1.189)	0.444
3~4	0.972 (0.938-1.008)	0.128	0.93 (0.858-1.007)	0.074	1.031 (0.88-1.207)	0.708
5~6	0.911 (0.856-0.969)	0.003	0.931 (0.814-1.064)	0.294	0.95 (0.723-1.248)	0.712
Daily	0.97 (0.929-1.012)	0.159	1.015 (0.929-1.109)	0.749	1.146 (0.97-1.353)	0.109
Period from menopause to inclusion (years)						
5~9	1.082 (1.049-1.117)	<0.001	1.042 (0.971-1.119)	0.253	1.28 (1.104-1.484)	0.001
10~	1.166 (1.114-1.219)	<0.001	1.235 (1.12-1.362)	<0.001	1.429 (1.176-1.737)	<0.001

BMI, Body mass index; CCI, Charlson comorbidity index; CI, confidence interval; HR, hazard ratio; MHT, menopausal hormone therapy; SES, socioeconomic status

[a] HRs were adjusted for age group, body mass index, socioeconic status, region, Charlson comorbidity index, parity, age at menarche, age at menopause, smoking, alcohol, physical exercise, period from menopause to inclusion.
