# Supplementary material for: Menopausal hormone therapy increases the risk of gallstones: Health Insurance Database in South Korea (HISK)-based cohort study
Source: PLoS One. 2023 Dec 4;18(12):e0294356. doi: 10.1371/journal.pone.0294356 (PMC10695378; doi:10.1371/journal.pone.0294356)
Supplement: S3 Table — (PDF) [file pone.0294356.s003.pdf]

Supplementary Table 3. The gallbladder disease cases per 100,000 person-years in this research

	Non-MHT	Tibolone	Combined Estrogen plus progestin by the manufacturer	Oral Estrogen	Combined Estrogen plus progestin by the physician	Topical estrogen
Total	40,037/11,602,949 (345)	9,572/2,423,410 (395)	5,237/1,550,042 (338)	2,619/696,521 (376)	309/85,479 (361)	128/27,520 (465)
Age at inclusion (years)						
40~49	3,029/1,097,318 (276)	1,507/434,404 (347)	1,124/359,727 (312)	622/187,708 (331)	50/15,298 (327)	30/5,966 (503)
50~59	17,315/5,608,350 (309)	6,129/1,574,393 (389)	3,458/1,019,662 (339)	1,425/384,832 (370)	184/50,420 (365)	79/16,798 (470)
60~69	13,329/3,530,851 (378)	1,704/371,545 (459)	584/158,256 (369)	469/103,705 (452)	67/17,428 (384)	18/4,342 (415)
70~	6,364/1,366,429 (466)	232/43,069 (539)	71/12,397 (573)	103/20,276 (508)	8/2,334 (343)	1/415 (241)
BMI (kg/m2)						
<18.5	604/207,319 (291)	152/40,377 (376)	84/29,984 (280)	37/9,906 (374)	8/1,731 (462)	2/569 (352)
18.5-22.9	11,484/3,895,178 (295)	3,468/959,904 (361)	2,085/688,496 (303)	816/253,646 (322)	131/35,061 (374)	39/10,259 (380)
23-24.9	10,187/3,040,954 (335)	2,590/671,926 (385)	1,412/416,741 (339)	717/194,342 (369)	79/23,771 (332)	36/7,306 (493)
25-29.9	14,356/3,783,310 (379)	2,935/669,267 (439)	1,441/374,226 (385)	900/210,027 (429)	78/22,436 (348)	49/8,428 (581)
≥30	2,288/481,311 (475)	308/60,291 (511)	162/30,483 (531)	124/23,431 (529)	12/1,872 (641)	1/802 (125)
SES						
Mid~high SES	37,691/11,155,205 (338)	9,107/2,343,413 (389)	5,068/1,513,589 (335)	2,537/678,998 (374)	300/83,630 (359)	123/26,805 (459)
Low SES	2,346/447,744 (524)	465/79,998 (581)	169/36,453 (464)	82/17,523 (468)	9/1,849 (487)	5/715 (699)
Region						
Urban area	12,008/3,408,252 (352)	3,194/759,708 (420)	1,822/529,470 (344)	860/220,908 (389)	170/43,432 (391)	57/12,462 (457)
Rural area	28,029/8,194,696 (342)	6,378/1,663,703 (383)	3,415/1,020,572 (335)	1,759/475,613 (370)	139/42,048 (331)	71/15,058 (471)
CCI						
0	24,272/7,665,639 (317)	6,131/1,652,855 (371)	3,527/1,100,871 (320)	1,713/487,827 (351)	206/59,190 (348)	76/18,137 (419)
1	8,458/2,262,737 (374)	2,001/471,667 (424)	1,001/280,325 (357)	521/125,841 (414)	55/16,134 (341)	34/5,036 (675)
≥2	7,307/1,674,573 (436)	1,440/298,889 (482)	709/168,847 (420)	385/82,853 (465)	48/10,155 (473)	18/4,348 (414)
Parity (years)						
0 or not respond	8,064/2,340,501 (345)	1,715/426,228 (402)	772/227,738 (339)	605/157,417 (384)	57/18,601 (306)	26/6,363 (409)
1	2,096/635,435 (330)	831/198,817 (418)	476/152,206 (313)	191/51,031 (374)	23/5,910 (389)	14/2,014 (695)
2	24,010/6,996,270 (343)	6,106/1,561,400 (391)	3,549/1,046,868 (339)	1,530/415,365 (368)	193/51,746 (373)	75/16,254 (461)
≥3	5,867/1,630,743 (360)	920/236,965 (388)	440/123,230 (357)	293/72,709 (403)	36/9,223 (390)	13/2,889 (450)
Age at menarche (years)						
<13	7,002/2,136,579 (328)	1,475/369,362 (399)	822/231,533 (355)	528/131,709 (401)	52/16,503 (315)	26/5,156 (504)

	Non-MHT	Tibolone	Combined Estrogen plus progestin by the manufacturer	Oral Estrogen	Combined Estrogen plus progestin by the physician	Topical estrogen
≥13	32,776/9,395,623 (349)	8,011/2,032,484 (394)	4,384/1,308,097 (335)	2,061/555,286 (371)	256/68,161 (376)	99/22,011 (450)
Age at menopause (years)						
40-44	5,377/1,594,163 (337)	1,303/308,749 (422)	648/187,274 (346)	569/154,039 (369)	38/11,517 (330)	23/5,570 (413)
45-49	11,623/3,389,186 (343)	3,046/800,465 (381)	1,742/523,883 (333)	941/248,141 (379)	94/27,810 (338)	51/10,010 (510)
50-54	19,417/5,651,904 (344)	4,445/1,132,382 (393)	2,508/734,681 (341)	975/260,693 (374)	150/39,329 (381)	46/10,271 (448)
55-	3,620/967,696 (374)	778/181,815 (428)	339/104,203 (325)	134/33,648 (398)	27/6,825 (396)	8/1,670 (479)
Smoking						
Never	35,875/10,621,090 (338)	8,524/2,189,401 (389)	4,702/1,402,794 (335)	2,357/636,802 (370)	283/78,952 (358)	121/25,293 (478)
Past	431/106,839 (403)	167/38,763 (431)	88/26,521 (332)	48/9,067 (529)	5/1,066 (469)	1/391 (256)
Current	1,107/270,504 (409)	482/102,265 (471)	256/68,868 (372)	112/23,697 (473)	10/2,317 (432)	1/598 (167)
Alcohol (g/week)						
None	32,482/9,426,061 (345)	7,366/1,826,955 (403)	3,946/1,148,572 (344)	2,039/539,880 (378)	246/68,861 (357)	101/21,945 (460)
~2/week	4,338/1,400,517 (310)	1,577/445,268 (354)	964/309,080 (312)	434/118,207 (367)	46/12,516 (368)	21/4,223 (497)
3~6/week	500/158,817 (315)	217/57,282 (379)	128/40,053 (320)	50/12,222 (409)	4/1,129 (354)	1/399 (251)
Daily	230/62,960 (365)	80/18,846 (424)	35/10,931 (320)	13/5,001 (260)	2/526 (380)	1/122 (817)
Physical exercise (per week)						
None	24,451/7,102,848 (344)	5,516/1,372,761 (402)	3,030/888,649 (341)	1,472/398,264 (370)	162/46,698 (347)	60/13,842 (433)
1~2	6,186/1,870,389 (331)	1,739/453,619 (383)	983/297,980 (330)	485/130,935 (370)	65/16,384 (397)	28/5,833 (480)
3~4	3,432/1,043,552 (329)	1,036/274,412 (378)	626/179,156 (349)	305/75,365 (405)	35/10,955 (319)	18/3,890 (463)
5~6	1,102/340,642 (324)	323/89,431 (361)	165/58,548 (282)	89/23,732 (375)	16/3,173 (504)	5/1,138 (439)
Daily	2,440/708,086 (345)	620/155,814 (398)	274/83,891 (327)	179/46,451 (385)	23/5,719 (402)	14/1,829 (765)
Period from menopause to inclusion (years)						
<5	13,190/4,478,344 (295)	5,076/1,384,801 (367)	3,358/1,034,317 (325)	1,273/365,424 (348)	154/43,488 (354)	64/14,134 (453)
5~9	8,316/2,533,296 (328)	2,304/571,550 (403)	1,098/318,434 (345)	668/173,910 (384)	81/20,971 (386)	34/7,243 (469)
10~	18,531/4,591,308 (404)	2,192/467,059 (469)	781/197,291 (396)	678/157,187 (431)	74/21,020 (352)	30/6,143 (488)

BMI, Body mass index, CCI, Charlson comorbidity index; MHT, menopausal hormone therapy; SES, socioeconomic status

Data are expressed as the case/person-years (case/ 100,000 person-years).
