# Supplementary material for: Menopausal hormone therapy increases the risk of gallstones: Health Insurance Database in South Korea (HISK)-based cohort study
Source: PLoS One. 2023 Dec 4;18(12):e0294356. doi: 10.1371/journal.pone.0294356 (PMC10695378; doi:10.1371/journal.pone.0294356)
Supplement: S4 Table — (PDF) [file pone.0294356.s004.pdf]

Supplementary table 4. Statistical analysis about the risk of gallbladder disease in relation to age

MHT	GB disease (GB stone + cholecystitis) [a]	
	HR (95% CI) [a]	P-value
50~59 years		
Tibolone	1.26 (1.202-1.321)	<0.001
Combined Estrogen plus progestin by the manufacturer	1.123 (1.055-1.196)	<0.001
Estrogen	1.134 (1.033-1.245)	0.008
Combined Estrogen plus progestin by the physician	1.188 (0.955-1.477)	0.122
Topical estrogen	1.473 (1.022-2.121)	0.038
60~69 years		
Tibolone	1.065 (0.956-1.186)	0.251
Combined Estrogen plus progestin by the manufacturer	0.887 (0.737-1.067)	0.204
Estrogen	1.236 (1.051-1.454)	0.011
Combined Estrogen plus progestin by the physician	0.817 (0.5-1.335)	0.419
Topical estrogen	0.7 (0.225-2.171)	0.536
70 years~		
Tibolone	1.162 (0.874-1.544)	0.301
Combined Estrogen plus progestin by the manufacturer	1.339 (0.791-2.268)	0.278
Estrogen	1.085 (0.718-1.638)	0.699
Combined Estrogen plus progestin by the physician	0 (0-1.181E+94)	0.931
Topical estrogen	0 (0-3.55E+171)	0.961

CI, confidence interval; HR, hazard ratio

a HRs were adjusted for body mass index, socioeconic status, region, Charlson comorbidity index, parity, age at menarche, age at menopause, smoking, alcohol, physical exercise, gallbladder disease, hypertension, diabetes mellitus, stroke, period from menopause to inclusion.
